# Transferring social-cognitive theories from individuals to healthcare professionals: a useful but simplified lens on clinical practice

**DOI:** 10.3389/frhs.2026.1755950

**Published:** 2026-03-05

**Authors:** Per Nilsen

**Affiliations:** Department of Health, Medicine and Caring Sciences, Linköping University, Linköping, Sweden

**Keywords:** behaviour change, healthcare professionals, organizational context, social-cognitive theories, transferability

## Abstract

This conceptual analysis examines the transferability of social-cognitive theories from individual health behaviours to healthcare professionals’ clinical behaviours. Although these theories provide a robust framework for explaining intentional and deliberative actions, their application to professional contexts risks oversimplifying the complex realities of healthcare work. Clinical behaviour is shaped not only by individual attitudes, beliefs, and perceived control but also by organizational hierarchies, procedural constraints, professional identity, and moral responsibility. Through analysis of empirical examples, the paper delineates the conditions under which social-cognitive theories retain explanatory power and those where alternative perspectives, such as workflow engineering, relational coordination, habit theory, and moral psychology, might offer greater insight. The paper argues for an integrative approach that situates individual cognition within environmental, organizational, and ethical contexts. By doing so, it refines understanding of when and how social-cognitive frameworks can inform implementation efforts, and where complementary theoretical lenses are needed to capture the realities of clinical practice.

## 1 Introduction

Social-cognitive theories such as the Theory of Planned Behaviour (1), the Theory of Reasoned Action (2), and Social Cognitive Theory (3) have been widely used to explain and predict health-related behaviour. These theories are among the most frequently applied in lifestyle and health behaviour research (4) and were developed to capture how individuals form intentions and make behavioural decisions by weighing potential risks and benefits, responding to social and normative influences, and evaluating their perceived capability or control over the behaviour (5).

Because these mechanisms are central to intentional behaviour change, constructs from social-cognitive theories have been integrated into implementation science models and frameworks to explain and influence healthcare professionals' adoption, implementation, and sustainment of evidence-based practices (6–8). However, the transfer of these theories from individuals' health-related behaviours to healthcare professionals' clinical behaviours rests on the assumption that similar cognitive processes, such as intention formation, evaluation of outcomes, and perceived control, underlie both personal and professional actions. Yet the validity of this assumption may vary, given the social, organizational, and ethical complexities of clinical practice (9, 10).

This study uses a conceptual analysis to examine the transferability of social-cognitive theories from individual health behaviours to professional clinical contexts. Conceptual analysis systematically clarifies and refines the meaning, assumptions, and scope of theoretical constructs, assessing their coherence and applicability across domains (11). The purpose here is to assess the plausibility and limits of key assumptions underlying social-cognitive theories and to identify contextual factors that enhance or constrain their explanatory power in healthcare settings.

## 2 Why the same theories can be applied

Many of the core mechanisms proposed by social-cognitive theories operate similarly across behavioural domains, whether the focus is on personal health behaviours or healthcare practitioners’ clinical behaviours. Within the Theory of Planned Behaviour, attitudes towards a behaviour (e.g., the perceived utility of a clinical guideline), subjective norms (what respected peers or supervisors are believed to do or expect), and perceived behavioural control (confidence in one's skills or the availability of resources) jointly influence intention, which predicts behaviour (1). Similarly, Social Cognitive Theory highlights self-efficacy, i.e., the belief in one's capability to perform a task (e.g., a new clinical procedure), and outcome expectancies (e.g., improved patient health) as key influences on behaviour, with observational learning (e.g., junior clinicians observing senior role models) playing a particularly strong role in professional settings (3).

Intentional, deliberative behaviours clearly exist within healthcare practice. Many professional decisions, such as the selection of antibiotics, ordering thromboprophylaxis, or the decision to offer vaccination, require cognitive evaluation and professional judgement. Therefore, they can change through the same pathways that influence personal health behaviours. In this sense, social-cognitive theories offer a valuable conceptual foundation for explaining and influencing professional practice (12, 13).

## 3 The case against uncritical application

Healthcare professionals' behaviours differ from personal health-related behaviours in both context and complexity (14, 15). They are socially structured and procedurally constrained within care pathways, protocols, and institutional workflows. Consequently, an individual's beliefs or intentions may be necessary but rarely sufficient for behaviour change. For instance, in antibiotic stewardship, a junior doctor may favour narrow-spectrum prescribing but be overruled by environmental pressures such as overcrowding, electronic defaults, or fear of patient or supervisor callbacks (16, 17). Thus, organizational and contextual forces often outweigh individual motivational factors such as attitude or perceived behavioural control.

Normative and organizational influences also operate in more formalized and coercive ways in healthcare than in personal contexts. Norms are codified through guidelines, audits, and power asymmetries and “subjective norms” often function as directives rather than mere social perceptions (18, 19). In the case of venous thromboembolism prophylaxis, for example, hospital policies often mandate structured risk assessments supported by pre-set order templates. Junior doctors may comply less out of personal endorsement of the intervention and more due to audit pressures and consultant expectations that leave little room for deviation. In this way, organizational norms and hierarchical authority supplant personal attitudes as the dominant drivers of behaviour (14, 20, 21).

Furthermore, autonomy and control are distributed differently in clinical environments. Social-cognitive theories assume that perceived behavioural control reflects individual confidence and capability, but in healthcare, it also depends on material and structural affordances, e.g., adequate staffing and equipment (22). Failures in behaviour often arise not from low self-efficacy but from resource constraints (23). Hand hygiene compliance offers a clear example. Whether or not a healthcare professional performs the behaviour depends less on beliefs about infection risk and more on the physical placement of dispensers, workflow interruptions, or restrictive glove policies.

In addition, healthcare professionals operate within dual motivational systems shaped by professional identity, moral duty, and fiduciary responsibility to patients (24–26). These motivations can override personal cost–benefit calculations that typically underpin social-cognitive theories. During end-of-life communication, for instance, clinicians may act from ethical commitment and empathy rather than from instrumental expectations about outcomes. Moral reasoning and affective engagement, which are dimensions underrepresented in standard cognitive theories, play a central role in guiding clinical practice (27).

Habitual and heuristic processes dominate much of everyday clinical practice. Under conditions of time pressure and cognitive load, healthcare professionals often rely on shortcuts such as ordering set defaults, heuristics, or well-rehearsed routines. Many actions are cue-driven rather than deliberate, reducing the relevance of constructs such as intention or perceived control (28–30).

## 4 When are social-cognitive theories least applicable?

Social-cognitive theories are least applicable when healthcare professionals' actions are shaped less by personal intention or perceived control and more by strict procedural rules (Table 1). In such highly protocolized settings, so-called strong situations marked by clear behavioural cues, firm incentives or sanctions, and little ambiguity (31), individual discretion is limited. For instance, peri-operative antibiotic administration is typically governed by anaesthetic protocols that leave little scope for independent judgement. In these contexts, frameworks from workflow engineering, checklist design, and reliability science could offer more useful lenses for understanding and improving performance (32, 33).

**Table 1:** Healthcare contexts and behavioural conditions in which social-cognitive theories have limited explanatory power, and alternative approaches may be more appropriate.

Healthcare contexts and behavioural conditions	Why are social-cognitive theories less applicable	Potentially more suitable theoretical or practical approaches
Highly protocolized tasks (e.g., peri-operative antibiotic timing)	Behaviour is governed by protocols, leaving minimal individual discretion or need for intentional decision-making	Workflow engineering, checklist design, reliability science, process control metrics
High interdependence across teams (e.g., implementing sepsis bundles across the emergency department, intensive care unit, and lab)	Behavioural success depends on coordination, communication, and shared understanding across professional groups, rather than individual intention	Team training, coordination tools, relational coordination theory, boundary-spanning roles
Dominance of environmental cues or habits (e.g., device sterilization, routine infection control)	Behaviour is automatic or environmentally cued rather than cognitively mediated	Nudge architecture, environmental redesign, habit formation frameworks, behavioural cue interventions
Rigid hierarchies and power asymmetries (e.g., escalating diagnostic disagreement)	Social and organizational hierarchies constrain open communication and individual agency	Speaking-up protocols, graded assertiveness training, culture and climate interventions
Ethically complex or emotionally charged encounters (e.g., goals of care or end-of-life discussions)	Behaviour is shaped by moral reasoning, emotion, and professional identity rather than deliberate intention	Reflective practice, ethics and communication training, Schwartz rounds, and moral distress assessment

The social-cognitive theories also have limited explanatory power when behaviours require high interdependence across multiple professional groups. Activities such as implementing sepsis bundles that span emergency, intensive care, and laboratory teams depend on coordination, shared mental models, and interprofessional communication. Here, theories such as relational coordination (34) and studies of communication in dynamic domains (35, 36) may offer better explanatory power.

When actions are dominated by environmental cues or habitual routines, as in device sterilization or routine infection control procedures, behaviour is more effectively explained through theories of behavioural economics and nudge design, environmental redesign, and habit formation rather than through conscious intention (37, 38). Similarly, rigid hierarchies can inhibit agency, making speaking-up protocols and culture change initiatives relevant (39, 40).

In ethically complex or emotionally charged encounters, such as discussions about goals of care or end-of-life decisions, behaviour may be shaped less by cognitive deliberation and more by emotional, moral, or identity-based processes (41, 42). These situations call for reflective practice, ethics and communication training, and emotional support mechanisms that recognize the moral and affective dimensions of clinical work (43).

## 5 Discussion

Social-cognitive theories continue to provide a valuable conceptual foundation for understanding intentional, deliberative behaviours in healthcare. Their emphasis on attitudes, norms, and perceived control explains many aspects of professional decision-making, particularly where behaviour is guided by reflection and individual agency. Constructs such as self-efficacy and outcome expectancy have informed practical strategies for healthcare professionals, ranging from communication training to audit and feedback. These contributions underscore the enduring relevance of cognitive approaches for influencing certain domains of clinical behaviour.

However, transferring these theories from individual to professional contexts risks simplification. Healthcare work is characterized by interdependence, hierarchy, and institutional constraint, conditions that differ from the autonomous, self-regulating settings in which these theories were developed. In such environments, individual cognition interacts with environmental, organizational, and moral structures that shape what is possible, not merely what is intended. Behaviour is therefore not only driven by personal attitudes or perceived control but also by distributed responsibility, resource availability, professional identity, and the ethical demands of patient care.

A further implication of this analysis is the need to conceptualize clinical behaviour as multi-level and temporally situated. Many actions unfold within sequences of decisions distributed across actors, time, and settings. What appears as an individual choice at one moment may, in fact, be the downstream product of earlier design decisions, resource allocations, and cultural expectations. Extending the discussion to incorporate temporal and system-level perspectives would reinforce the argument that cognition operates within structured environments. For example, an intervention that strengthens clinicians' intentions without addressing competing workflow demands or misaligned performance metrics may produce only transient change. A richer account of how structural conditions enable or constrain the translation of intention into action would deepen the explanatory framework proposed in this paper.

Recognizing these multi-level and temporally embedded dynamics establishes a more nuanced agenda for behavioural explanation in implementation science. Integrating insights from fields such as organizational psychology, relational coordination, behavioural economics, and moral psychology could create a more comprehensive understanding of how and why healthcare professionals act. Implementation strategies that align cognitive, environmental, and organizational levers are more likely to achieve sustained change than those targeting healthcare professionals' beliefs or intentions alone.

Moreover, greater attention could be given to the methodological implications for implementation research. If behaviour is shaped by interacting cognitive, organizational, and moral factors, then single-theory designs may inadequately capture the determinants of practice. Mixed-method approaches and multi-level analytic strategies may be better suited to disentangling how mechanisms operate under different contextual conditions. Explicitly linking theoretical integration to research design would strengthen the practical contribution of this work. It would also encourage researchers to move beyond asking whether a theory “works” in general, toward examining under what circumstances specific theoretical constructs meaningfully contribute to understanding and improving clinical practice.

In conclusion, social-cognitive theories provide valuable insight into the intentional dimensions of healthcare professionals' behaviour, yet applying them to clinical contexts inevitably reduces a more complex reality. Professional action is shaped as much by organizational systems, social structures, and moral commitments as by individual cognition and intention. A fuller understanding therefore, calls for a broader, integrative approach—one that extends social-cognitive perspectives through environmental, organizational, and ethical lenses to more accurately reflect how behaviour unfolds in practice.

## Data Availability

The original contributions presented in the study are included in the article/Supplementary Material, further inquiries can be directed to the corresponding author.
